# UPARANT is an effective antiangiogenic agent in a mouse model of rubeosis iridis

**DOI:** 10.1007/s00109-019-01794-w

**Published:** 2019-06-26

**Authors:** Filippo Locri, Massimo Dal Monte, Monica Aronsson, Maurizio Cammalleri, Mario De Rosa, Vincenzo Pavone, Anders Kvanta, Paola Bagnoli, Helder André

**Affiliations:** 1 Department of Clinical Neuroscience, Division of Eye and Vision, St Erik Eye Hospital, Karolinska Institutet, Polhemsgatan 50, 112 82 Stockholm, Sweden; 2 Department of Biology, University of Pisa, Pisa, Italy; 3 Department of Experimental Medicine, Second University of Naples, Naples, Italy; 4 Department of Chemical Sciences, University of Naples Federico II, Naples, Italy

**Keywords:** Rubeosis iridis, Inflammation, Antiangiogenic drug, UPARANT, Cenupatide

## Abstract

**Abstract:**

Puncture-induced iris neovascularization (rubeosis iridis; RI) in mice is associated with upregulation of extracellular matrix (ECM) degradation and inflammatory factors. The anti-angiogenic and anti-inflammatory efficacy of UPARANT in reducing RI was determined by noninvasive, in vivo iris vascular densitometry, and confirmed in vitro by quantitative vascular-specific immunostaining. Intravitreal administration of UPARANT successfully and rapidly reduced RI to non-induced control levels. Molecular analysis revealed that UPARANT inhibits formyl peptide receptors through a predominantly anti-inflammatory response, accompanied with a significant reduction in ECM degradation and inflammation markers. Similar results were observed with UPARANT administered systemically by subcutaneous injection. These data suggest that the tetrapeptide UPARANT is an effective anti-angiogenic agent for the treatment of RI, both by local and systemic administrations. The effectiveness of UPARANT in reducing RI in a model independent of the canonical vascular endothelial growth factor (VEGF) proposes an alternative for patients that do not respond to anti-VEGF treatments, which could improve treatment in proliferative ocular diseases.

**Key messages:**

- UPARANT is effective in the treatment of rubeosis iridis, both by local and systemic administrations.
- UPARANT can reduce VEGF-independent neovascularization.

**Electronic supplementary material:**

The online version of this article (10.1007/s00109-019-01794-w) contains supplementary material, which is available to authorized users.

## Introduction

In the eye, the vasculature plays a key role in detecting light and supplying oxygen and nutrients. Vascular networks and blood vessel numbers are precisely established from development to adulthood, ranging from the avascular cornea and lens for transparency, the fractal retinal vasculature for light sensing, to the highly vascularized uvea for oxygen supply. The uvea includes the iris, ciliary body, and choroid. Iris vasculature originates from the outer uveal limbal limits and is characterized by numerous anastomoses between arteries and veins. This peculiar vascular architecture allows iris blood vessels to supply oxygen and nutrients to the anterior segment and maintain corneal and lens homeostasis [1]. Angiogenesis, the formation of new blood vessels from the existing vascular bed, is fundamental in various physiological processes, including development and wound healing. Angiogenesis is finely regulated by various factors, such as vascular endothelial growth factor (VEGF), the plasminogen-activator system, and inflammatory factors. Imbalances in stimulatory and inhibitory factors can lead to pathologic angiogenesis [2], as is the case in sight-threatening ocular diseases. Proliferative diabetic retinopathy (PDR) and central retinal vein occlusion (CRVO) are characterized by increased neovascularization and inflammation and correlate with pathologic rubeosis iridis (RI), the clinical term for excessive neovascularization in the iris. These conditions can culminate in sight-threatening neovascular glaucoma (NVG) [3, 4]. In the progression of proliferative retinopathies (PR), the imbalance of angiogenic and inflammatory factors in both the posterior and anterior chambers of the eye stimulates iris vasculature to undergo angiogenesis [5]. Rubeosis iridis obstructs the flow of aqueous humor through the trabecular meshwork, resulting in elevated intraocular pressure and ultimately NVG [6]. Pharmacological treatment of RI with anti-VEGF agents is becoming more established, albeit with some limitations, and the need for improved therapies has been suggested [7, 8].

UPARANT (previously known as UPARANT) belongs to a family of tetrapeptides which strongly inhibits endothelial cell migration by interfering with the complex crosstalk activation of formyl peptide receptors (FPR) [9–11]. UPARANT administration was shown to be effective in counteracting angiogenesis and ameliorating visual dysfunction in rodent models of oxygen-induced retinopathy (OIR) [12], choroidal neovascularization (CNV) [13], and diabetic retinopathy (DR) [14, 15].

An in vivo mouse model of puncture-induced RI has been established [16, 17]. This model was characterized by a wound-healing response displaying increased expression of the plasminogen activator and inflammation systems as angiogenesis factors. It allows for direct, noninvasive quantification of the iris vasculature. Additionally, the model undergoes neovascularizarion independently of the canonical VEGF signaling, which renders the puncture-induced RI a unique model for angiogenic studies [16]. In this context, the anti-angiogenic efficacy of intravitreal UPARANT administration in counteracting the iris neovascular response has been evaluated. The effects of UPARANT on angiogenesis and inflammation markers characteristic of the model were subsequently determined following systemic administration, where UPARANT displayed marked benefits in mitigating neovascularization in the puncture-induced mouse model of RI.

## Materials and methods

### Animals

Twenty-three 12.5-day-old (P12.5) BALB/c mice of either sex (Charles River, Cologne, Germany) were used in accordance with the statement for the Use of Animals in Ophthalmologic and Vision Research and the European Communities Council directive for animals’ use for scientific purposes, and the study protocols were approved by Stockholm’s Committee for Ethical Animal Research. Mice were housed in litters with a nursing mother on a 12-h day/night cycle, with free access to food and water, and monitored daily. Euthanasia was performed by cervical dislocation, as approved by the ethical committee.

### Pharmacological treatment

UPARANT, designated cenupatide (CAS number: 1006388-38-0) by the World Health Organization–assigned international non-proprietary name [10, 18], was dissolved in sterile phosphate-buffered saline (PBS; ThermoFisher Scientific Inc., Waltham, MA, USA) in the form of succinate salt at a concentration of 10 g/L for intravitreal injection, and 20 mg/kg for subcutaneous administration (7.6 g/L and 15.2 mg/kg of active pharmaceutical ingredient, respectively), as suggested previously [13] and adjusted to mouse pups body weight.

### Puncture-induced RI

**Fig. 1:**
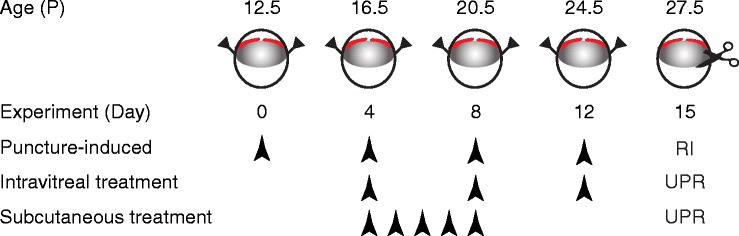
Scheme of animal models and treatments. Mouse pups (P12.5–24.5) were subjected to two uveal punctures on opposing sites of the eye. Punctures were repeated every fourth day (arrowheads; RI), from experimental days 0 through 12. Intravitreal treatments with 7.6 g/L UPARANT (UPR) were carried out at days 4, 8, and 12. UPARANT subcutaneous treatment with 15.2 mg/kg was performed as a daily loading dose during days 4 through 8

Mouse pups (across 4 litters), anesthetized with 4% isoflurane (Baxter, Kista, Sweden) in room-air, were subjected to uveal punctures on both eyes, as previously described [16, 17]. Briefly, puncture procedure to induce the RI model was performed every 4 days until experimental day 12. The procedures consisted of two self-sealing uveal punctures with a 30G beveled needle immediately posterior to the limbus. Intravitreal administrations of 1 μL of UPARANT solution were performed on experimental days 4, 8, and 12 on one eye of 12 mice, while the fellow-eye was left untreated. One additional group of six mice was kept as non-punctured control. Finally, five mice were subjected to RI protocol in one eye, leaving the fellow-eye as non-punctured control. On experimental day 4, mouse pups received subcutaneous administrations of UPARANT solution daily until experimental day 8 (5 days loading dose). Figure 1 summarizes the animal models and treatment schemes. After each procedure, mice were treated with a drop of 1% tetracaine hydrochloride solution (Bausch & Lomb, Rochester, NY, USA), rehydrated with a subcutaneous injection of sterile saline solution (9 g/L NaCl; B. Braun, Melsungen, Germany), and returned to the nursing mother in a clean cage. On experimental day 15, mice were euthanized. Eyes were carefully dissected and cleared from extraneous tissues, rinsed in PBS, and either frozen in liquid nitrogen and stored at − 80 °C for molecular analysis, or fixed for 6 h in 4% buffered formaldehyde (FA; Solveco, Rosersberg, Sweden) for immunofluorescence.

### Quantitative noninvasive in vivo iris vasculature analysis

Prior to puncture or intravitreal injection on each experimental day, irises were photographed using an objective adapted camera (Apple Inc., Cupertino, CA, USA) for the surgical stereoscope (Wild M650; Wild, Heerbrugg, Switzerland). Whole-irises were selected as region of interest (ROI) from the in vivo photos and converted to 8-bit to enhance vascular structures over background. Vascular density was analyzed by densitometry using the ImageJ software (NIH freeware), corrected to total area of irises, and presented as percentage of control.

### Quantitative immunofluorescence

Irises from FA-fixed eyes were carefully dissected from the whole-eye and processed for free-floating immunofluorescence, as previously described [19]. Antibodies used are summarized in Table 1. Images were acquired by fluorescence microscopy using an Axioskop 2 plus with the AxioVision software (Zeiss, Gottingen, Germany). Quantitative analysis of iris vascular networks was performed with the AngioTool software [20]. Analyses of total vessel length as a correlation of the total vasculature, endpoints which represent sprouts, and number of junctions as a measure of vascular branching were performed on magnification panels of the whole-iris, corrected to tissue area, and expressed as percentage of control.

**Table 1:** List of antibodies

Primary antibody	Host	Dilution	Application	Source	Cat. no.
Anti-actin	Rabbit	1:1000	WB	Sigma-Aldrich Corp., St. Louis, MO, USA	A2066
Anti-CD31	Rat	1:200	IF	BD Biosciences, Bedford, MA, USA	562939
Anti-CREB	Rabbit	1:200	WB	Santa Cruz Biotechnology, Santa Cruz, CA, USA	sc-25785
Anti-CREB pSer133	Goat	1:200	WB	Santa Cruz Biotechnology	sc-7978
Anti-CXCR4	Rabbit	1:200	WB	Bio-Techne Corp., Abingdon, UK	NB100-56437
Anti-FPR1	Goat	1:200	IF	Santa Cruz Biotechnology	sc-13198
Anti-FPR2	Rabbit	1:200	IF	Santa Cruz Biotechnology	sc-66901
Anti-FPR3	Rabbit	1:200	IF	Santa Cruz Biotechnology	sc-66899
Anti-HIF-1α	Rabbit	1:200	WB	Bio-Techne Corp.	NB100-134
Anti-IL6	Rabbit	1:200	WB	ABCam, Cambridge, UK	ab6672
Anti-MMP2	Rabbit	1:200	WB	Bio-Techne Corp.	NB200-193
Anti-NFκB	Rabbit	1:200	WB	Santa Cruz Biotechnology	sc-372
Anti-NFκB pSer276	Rabbit	1:200	WB	Santa Cruz Biotechnology	sc-101749
Anti-VEGF	Rabbit	1:200	WB	ABCam	ab9570
Secondary antibodies	Host	Dilution	Application	Source	Cat. no.
Anti-goat-CF647	Donkey	1:500	IF	Sigma-Aldrich Corp.	SAB4600175
Anti-goat-HRP	Donkey	1:2000	WB	ThermoFisher Scientific Inc	A15999
Anti-rabbit-A647	Goat	1:500	IF	ThermoFisher Scientific Inc	A21245
Anti-rabbit-HRP	Swine	1:2000	WB	Dako, Carpinteria, CA, USA	P0399
Anti-rat-A546	Goat	1:500	IF	ThermoFisher Scientific Inc.	A11006

*WB*, western blot; *IF*, immunofluorescence; *A*, Alexa fluorophore; *HRP*, horse-radish peroxidase; *CF*, biotium fluorophore

### Quantitative PCR

Liquid nitrogen frozen eyeballs were processed using an RNA isolation and purification kit (Qiagen, Hilden, Germany) following the manufacturer’s recommendations. Total RNA (1 μg) was retrotranscribed to cDNA, and gene expression was analyzed by quantitative PCR (qPCR), as previously described [21]. Expression levels were determined by relative transcript expression to two housekeeping genes (TATA-box binding protein, TBP; and hypoxanthine guanine phosphoribosyl transferase, HRPT) and normalized to non-punctured controls (ΔΔCT method). All PCR reagents, PrimePCR primer-pairs (Table 2), and equipment were from BioRad Laboratories, Hercules, CA, USA.

**Table 2:** List of primer-pairs

Gene	Design	Cat. no.
CCL2	Exonic	qMmuCED0048300
CXCR4	Exonic	qMmuCED0026325
EPO	Exonic	qMmuCED0047041
FPR1	Intron-spanning	qMmuCID0015439
FPR2	Exonic	qMmuCED0037749
FPR3	Exonic	qMmuCED0040524
HPRT	Intron-spanning	qMmuCID0005679 (HK)
IL1β	Intron-spanning	qMmuCID0005641
IL6	Intron-spanning	qMmuCID0005613
MMP2	Intron-spanning	qMmuCID0021124
MMP9	Intron-spanning	qMmuCID0021296
PKG1	Exonic	qMmuCEP0062122
PLGF	Intron-spanning	qMmuCID0017000
PAI-1	Intron-spanning	qMmuCID0012875
TBP	Intron-spanning	qMmuCID0040542 (HK)
TGFα	Intron-spanning	qMmuCID0006309
TGFβ	Exonic	qMmuCED0044726
uPA	Intron-spanning	qMmuCID0022420
uPAR	Intron-spanning	qMmuCID0017011
VEGF	Exonic	qMmuCED0040260
VEGFR1	Intron-spanning	qMmuCID0016762
VEGFR2	Intron-spanning	qMmuCID0005890

*HK*, housekeeping gene

### Western blot analysis

Whole-eye protein extracts were prepared by homogenization in CelLytic-MT (Sigma-Aldrich Corp) [19] supplemented with a phosphatase and protease inhibitor cocktail (Roche, Mannheim, Germany). Samples were quantified by the Bradford method (BioRad Laboratories), and 15 μg of total protein was separated by SDS-PAGE and transblotted onto polyvinylidene difluoride (PVDF) membranes. Immunoblots were performed as previously described [19] with selected primary and secondary antibodies (Table 1). Blots were developed with Clarity Western enhanced chemiluminescence reagent (BioRad Laboratories). Images were acquired on a ChemiDoc XRS^+^ (BioRad Laboratories), and protein levels were determined by densitometry analysis using the Image Lab 3.0 software (BioRad Laboratories). Protein levels were corrected to the actin loading control or non-phosphorylated proteins, when appropriate.

### Statistical analysis

Noninvasive in vivo iris vasculature analyses were conducted for 8 mice per group (*n* = 8 eyes) by two-way ANOVA with Bonferroni posttest. Remainder experiments were performed on 4 mice per intravitreal group (*n* = 4 eyes) or 5 mice for subcutaneous administrations (*n* = 5 eyes). Analysis was performed by one-way ANOVA with Bonferroni posttest (*p* < 0.05 was considered significant).

## Results

### UPARANT mitigates uveal puncture-induced iris neovascularization

The cornea’s transparency and the deficiency of pigmentation in albino BALB/c mice enable in vivo noninvasive analysis and the quantification of iris blood vessels during the experimental procedure. The efficacy of intravitreally administered UPARANT on iris macrovascular responses in the RI mouse model is evaluated (Fig. 2a). Densitometry analysis of in vivo iris vasculature demonstrated a significant increase (*p* < 0.001) of approximately 20% in blood vessel density 4 days post-induction in RI eyes, as compared with the control group (Fig. 2b). Intravitreal injection of UPARANT caused a regression of vessel density to control levels at experimental day 8 (*p* < 0.001 versus RI), a result that was sustained through the study’s protocol (Fig. 2b).

**Figure:**
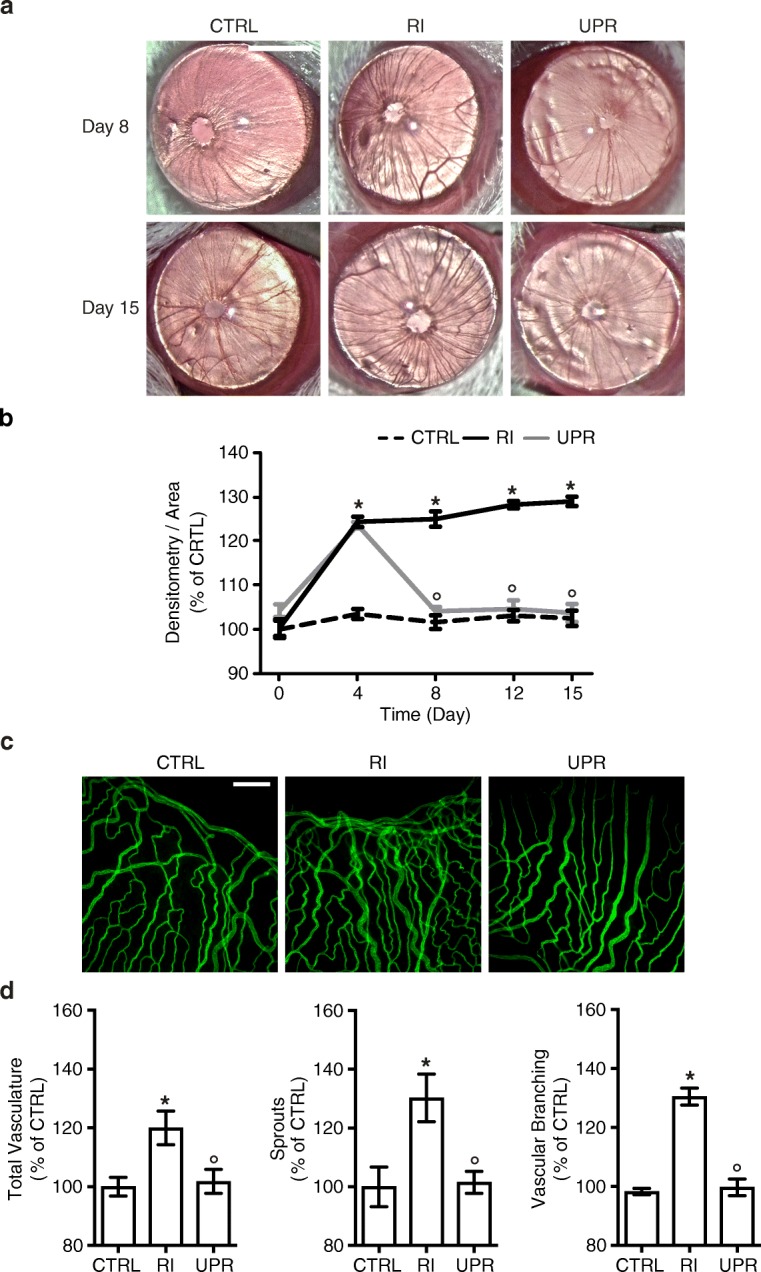

Subsequently, immunofluorescence assays with cluster of differentiation (CD)31 as an endothelial marker were performed on irises on day 15 (Fig. 2c), and the microvascular bed was analyzed for total vasculature, sprouts, and vascular branching using a vasculature-specific software [20]. Microvasculature parameters of RI eyes (Fig. 2d) displayed a significant increase of approximately 30% (*p* = 0.035, total vasculature; *p* = 0.028, sprouts; *p* < 0.001, branching) compared with non-punctured controls. Intravitreal UPARANT administration reduced the all analyzed microvascular parameters to control levels and significantly different from RI eyes (*p* = 0.049, total vasculature; *p* = 0.036, sprouts; *p* < 0.001, branching).

### UPARANT modulates FPR1 expression in iris vasculature

**Fig. 3:**
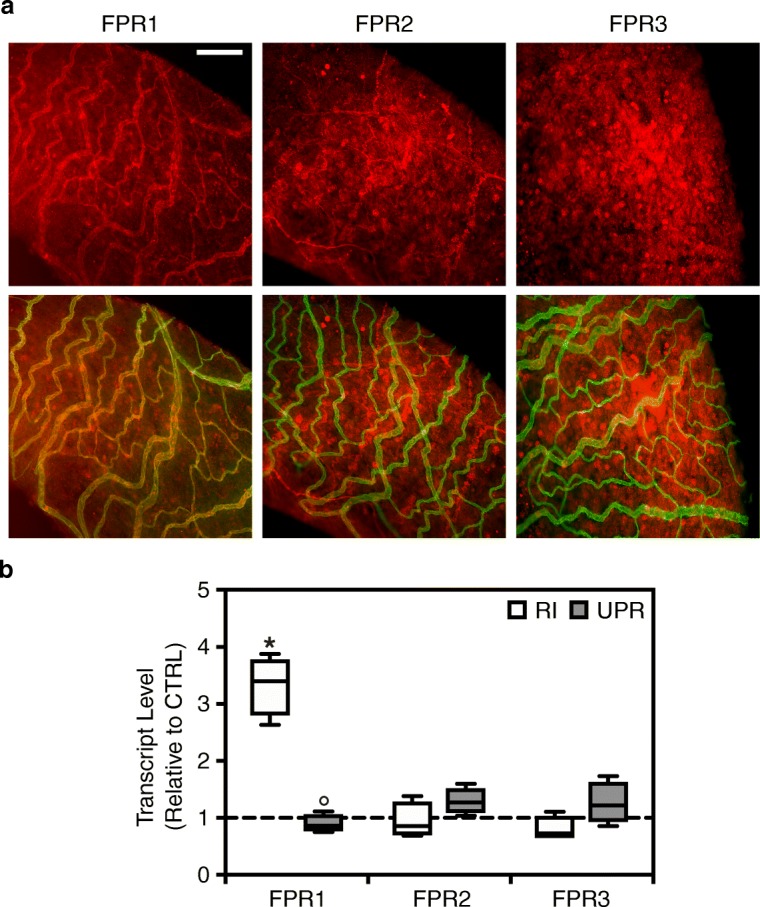
UPARANT acts through FPR1 signaling in iris neovasculature. **a** Representative images of iris vasculature co-immunostained for CD31 (green), and FPR1, FPR2, or FPR3 (red). Scale bar = 200 μm. **b** Transcript levels of FPRs were quantified by qPCR on non-punctured control (CTRL) eyes, RI-induced eyes, and 7.6 g/L UPARANT (UPR)-treated eyes by intravitreal administration. Data is represented as box plots of independent eyes per group (*n* = 4), and normalized to CTRL. Statistical analysis was performed by one-way ANOVA with Bonferroni posttest (*p* < 0.05: * vs. CTRL, ° vs. RI)

Previous studies have shown that UPARANT’s anti-angiogenic effects are exerted through the modulation of FPRs signaling [10, 11, 13, 15]. To investigate the potential target of UPARANT on iris vasculature, FPR1, -2, and -3 were co-immunostained with CD31 (Fig. 3a). Immunostained irises displayed a strong colocalization of FPR1 with the iris vasculature, while FPR2 and -3 showed a less intense colocalization signal. Subsequently, gene expression analysis was performed by qPCR to assess UPARANT’s effects on FPR expression. In RI eyes (Fig. 3b), an approximate 3.5-fold increase in FPR1 transcript levels was observed compared with controls (*p* < 0.001). Intravitreally administered UPARANT reduced FPR1 overexpression to control levels, and FPR levels were significantly lower when compared with RI eyes (*p* < 0.001). Notably, no alteration in transcript levels of FPR2 or -3 was observed.

### UPARANT reduces transcriptional activation of pro-inflammatory factors in the rubeosis iridis model

**Fig. 4:**
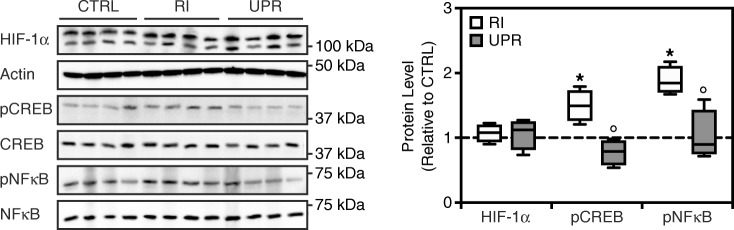
UPARANT reduces transcriptional activation of CREB and NFκB in iris neovascularization. Representative western blots of HIF-1α, CREB, and NFκB, of non-punctured control (CTRL), RI-induced, and treated eyes with 7.6 g/L UPARANT (UPR) administered intravitreally. Actin was used as the loading control. Densitometry analysis of protein levels was corrected versus actin or corresponding non-phosphorylated protein. Box plots of independent eyes per group (*n* = 4) represent quantitative data normalized to CTRL. Statistical analysis was performed by one-way ANOVA with Bonferroni posttest (*p* < 0.05: * vs. CTRL, ° vs. RI)

The effects of UPARANT have been associated previously with transcription factors involved in angiogenesis, particularly transcriptional activators mediating hypoxia and pro-inflammatory responses [12, 13]. Expression levels of hypoxia-inducible factor (HIF)–1α, cyclic AMP response element–binding protein (CREB), nuclear factor (NF)κB, and phosphorylated CREB and NFκB represented the prevalence of master regulators of the hypoxia and inflammation responses, and were assayed by immunoblotting (Fig. 4). No HIF-1α response was observed in the RI mouse model, nor upon treatment with UPARANT. A significant increase of phosphorylated CREB (*p* = 0.029) and NFκB (*p* = 0.030) protein levels versus control was observed in RI eyes. Intravitreal UPARANT–treated eyes showed a statistically significant decrease of both CREB and NFκB protein phosphorylation levels compared with RI (*p* = 0.002, CREB; *p* = 0.036, NFκB).

### UPARANT downregulates inflammation and extracellular matrix degradation markers associated with iris neovascularization

**Fig. 5:**
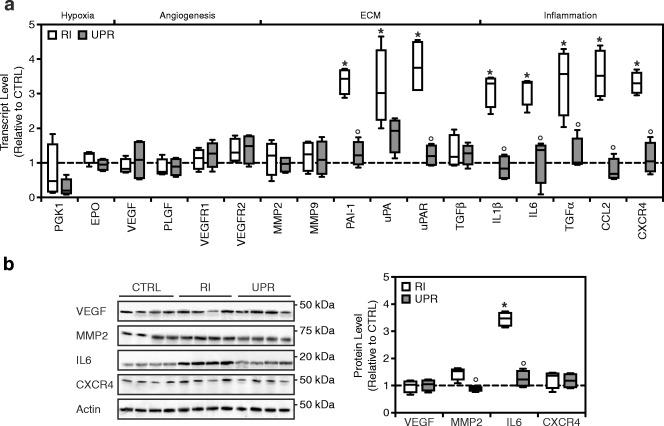
UPARANT counteracts inflammation and extracellular matrix degradation in iris neovascularization. **a** Transcript levels of genes involved in hypoxia response, canonical angiogenesis, ECM degradation, and inflammation were quantified by qPCR on non-punctured control (CTRL), RI-induced, and 7.6 g/L UPARANT (UPR) intravitreally treated eyes. Data are presented as box plots of independent eyes per group (*n* = 4), and normalized to CTRL. **b** Representative western blots of VEGF, MMP2, IL-6, and CXCR4, and the loading control actin for CTRL eyes, RI-induced eyes, and UPR-treated eyes by intravitreal administration. Densitometry analysis of protein levels is corrected versus the loading control and results are presented normalized to CTRL as box plots of independent eyes per group (*n* = 4). Statistical analysis was performed by one-way ANOVA with Bonferroni posttest (*p* < 0.05: * vs. CTRL, ° vs. RI)

Iris neovascularization in mice has been associated with increased expressions of inflammation and extracellular matrix (ECM) degradation markers [16]. To assess UPARANT effects on the expression of markers associated with iris neovascularization, a qPCR assay was performed. Eyes with RI displayed a significant increase in ECM degradation and inflammation markers (Fig. 5a). Levels of plasminogen-activator inhibitor (PAI)-1 (*p* < 0.001), urokinase-like plasminogen activator (uPA; *p* = 0.004), uPA receptor (uPAR; *p* < 0.001), interleukin (IL)-1β (*p* < 0.001), IL-6 (*p* = 0.019), transforming growth factor (TGF)α (*p* < 0.001), chemokine C-C motif ligand (CCL)2 (*p* = 0.001), and chemokine C-X-C motif receptor (CXCR)4 (*p* = 0.004) were significantly increased compared with the controls. Intravitreal UPARANT administration reduced the overexpression of these markers to control levels. Lastly, genes involved in the hypoxia response (phosphoglycerate kinase (PGK1) and erythropoietin (EPO)) and canonical angiogenesis (VEGF, placental growth factor (PLGF), and their receptors) did not appear to be regulated in the mouse RI model. Similar findings were observed for TGFβ.

To illustrate protein expression patterns of angiogenic, ECM degradation, and inflammation markers, immunoblotting was performed for VEGF, matrix metalloproteinase (MMP)2, IL-6, and CXCR4 on non-punctured control, RI, and intravitreal UPARANT–treated eyes (Fig. 5b). In the RI model, no statistically significant increase in expression of VEGF was observed, confirming the absence of canonical VEGF stimulation on iris neovasculature from the gene expression analysis. A statistically significant increased expression of IL-6 (*p* < 0.001) was observed in RI eyes, which were reduced to control levels by intravitreal administration of UPARANT (*p* < 0.001). Interestingly, MMP2 and CXCR4 levels were not statistically increased versus controls in RI eyes. However, intravitreal treatment with UPARANT significantly reduced MMP2 levels when compared with RI-induced eyes (*p* = 0.036).

### Systemic administration of UPARANT is effective in reducing iris neovascularization

**Fig. 6:**
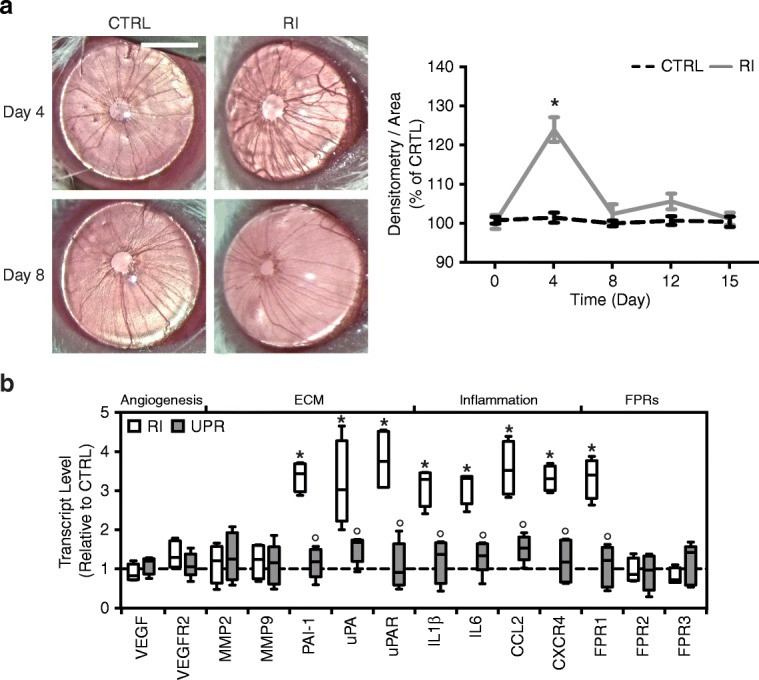
Effectiveness of UPARANT in mitigating iris neovascularization upon subcutaneous administration. **a** Noninvasive in vivo RI-induced iris vasculature upon subcutaneous treatment with 15.2 mg/kg UPARANT was analyzed by densitometry, normalized to percentage of non-punctured fellow-eye control (CTRL), and presented as mean ± SEM of independent eyes (*n* = 5 per group). Statistical analysis was performed by two-way ANOVA with Bonferroni posttest. Pictures illustrate mouse eyes at experimental days 4 and 8 of the corresponding groups treated with subcutaneous UPARANT. Scale bar = 1 mm. **b** Transcript levels were quantified by qPCR on RI-induced eyes treated subcutaneously with vehicle (*n* = 4 per group) or with UPARANT (UPR; *n* = 5 per group). Results were normalized to CTRL and presented as box plots. Statistical analysis was performed by one-way ANOVA with Bonferroni posttest (*p* < 0.05: * vs. CTRL, ° vs. RI)

To assess the potential efficacy of subcutaneous treatment with UPARANT in mitigating iris neovascularization, one eye of each mouse pup was induced with RI, while the fellow-eye was kept non-punctured as control. On experimental day 4 (Fig. 6a), RI-induced eyes displayed a significant increase of over 25% in vascularization compared with controls (*p* < 0.001). After a 5-day loading period with subcutaneous injections, between experimental days 4 and 8, UPARANT effectively counteracted the iris vascular response. No statistical difference was determined between the induced eyes and fellow controls immediately after the last subcutaneous injection and for the duration of the protocol, as determined by in vivo noninvasive iris vascular densitometry (Fig. 6a). On experimental day 15, qPCR analysis of UPARANT-treated eyes was performed and compared with RI eyes treated with subcutaneous saline, and the results directly paralleled the findings for intravitreal administration. In the RI model (Fig. 6b), ECM degradation (PAI-1, uPA, uPAR) and inflammation (IL-1β, IL-6, CCL2, CXCR4) markers were reduced to control levels upon subcutaneous administration of UPARANT and were statistically lower when compared with vehicle-treated animals (*p* < 0.001 on all). As before, VEGF signaling was not regulated in this model, and FPR1 transcript levels were significantly reduced compared with the vehicle-treated groups (*p* < 0.001).

## Discussion

In this study, the tetrapeptide UPARANT is shown to be effective in mitigating RI, both by intravitreal and subcutaneous administrations, which presents the first evidence of pharmacological treatment in the small rodent model of RI. In this model, iris neovascularization is induced by uveal punctures and is associated with mechanisms of wound healing, while independent of VEGF signaling [16, 17]. In contrast, patients with RI generally present elevated VEGF levels in the eye due to PR [4], and clinical management of RI through anti-VEGF drugs has become more established [7]. Nevertheless, some patients do not respond to anti-VEGF treatments [8], which has been correlated to VEGF-independent mechanisms of pathological neovascularization [22]. In this context, the VEGF-independent mouse model of iris neovascularization appears to be of particular interest in assessing future pharmacological substances, such as UPARANT for the treatment of neovascular diseases for patients that refract to anti-VEGF therapies, which consequently highlights a role for UPARANT in reducing pathological vasculature independently of the canonical neoangiogenesis pathways.

Intravitreal UPARANT treatment rapidly reduced RI macrovasculature. During murine post-partum development, the iris vasculature matures through arterial to venous anastomosis [23], where blood vessel sprouting and branching are hallmarks. Analysis of irises with vascular-specific immunofluorescence reveals that the microvasculature of RI treated with UPARANT is indistinguishable from controls in the relative number of blood vessels, number of sprouts, and branching index. Interestingly, the newly formed vessels did not display neovascular leakage in the mouse model of RI (Suppl. Fig. 1), which could be associated with vascular remodeling of iris anastomoses rather than the canonical sprouting angiogenesis, as previously suggested [16]. In general, UPARANT displays a fast and broad efficacy in reducing macro- and microvascular pathologic events related to the RI model.

The mechanisms of action of UPARANT are to date somewhat elusive. Nevertheless, studies have demonstrated an antagonistic effect of UPARANT on FPR signaling [10, 11, 13, 15], with putative affinity to all three receptor orthologs. Consequently, FPR expression was determined in the mouse iris for the first time. FPR1 is readily detected in the iris tissue with a clear vascular colocalization, while FPR2 and -3 display lower staining intensity and weaker vascular localization. These findings indicate a predominant expression of FPR1 in iris endothelial cells and are supported by a strong induction of FPR1 expression in RI-induced eyes. Interestingly, UPARANT treatment reduced FPR1 overexpression in RI eyes, suggesting a mode of action through FPR1 signaling in the mouse models of RI.

Activation of FPRs in animal models has been associated with hypoxia and inflammation pathways [13, 14, 18]. Analysis of transcription factors HIF-1α, CREB, and NFκB, as master regulators of the hypoxia and pro-inflammatory cellular responses, suggests that UPARANT inhibition of FPR1 in the induced RI mouse model is predominantly mediated through inflammation pathways and independent of hypoxia signaling. In fact, evaluations revealed that transcripts and proteins associated with hypoxia and canonical angiogenesis were not upregulated in the model of RI.

Activation of CREB and NFκB, through upregulation of the plasminogen-activator system and inflammatory cytokines, plays a pivotal role in angiogenesis [24–28]. In agreement with the activity of UPARANT on phosphorylation levels of both CREB and NFκB, transcript levels of genes associated with inflammation, ECM regulation, and ECM degradation are downregulated to control levels in the mouse model of RI. The effects of UPARANT on protein expression levels in the mouse model of RI further contribute to ECM degradation and inflammation-mediated pathways. The inflammatory cytokine IL-6 is increased in RI-induced eyes and readily reduced by UPARANT treatment. In addition, the protein levels of MMP2 are discretely elevated and reduced by UPARANT treatment. Molecular interpretation of the elevated transcripts and IL-6 and MMP2 protein levels in the RI mouse model could be biased due to use of whole-eye, rather than isolated irises. The particularity of the uveal puncture-induced iris neovascularization model assumes molecular communication between the posterior segment of the eye, namely the uvea and vitreous body, with responses observed in the iris within the anterior segment of the eye [16, 21]. As such, using whole-eye tissues benefits molecular analysis of RI-induced eyes and subsequent intravitreal injections of UPARANT by granting a wider perspective between both compartments of the eye involved in this mouse model.

Subcutaneous UPARANT administration has been shown to reach ocular tissues with pharmacological safety and reduce CNV lesions in a laser-induced murine model [13]. In a rat model of diabetes, systemic administration of UPARANT restored the blood retinal barrier and recovered electroretinogram [14]. In the present mouse model of RI, systemic administration of UPARANT decreases iris neovasculature and downregulates ocular transcripts to control levels. The results of systemic administration mimic those of intravitreal administration. Collectively, these findings are in agreement with previous studies that demonstrate inflammation- and ECM degradation–dependent yet VEGF-independent neovascularization of RI in the mouse model [16]. The results indicate that UPARANT can act on multiple pro-angiogenic pathways, in contrast to anti-VEGF treatments that are restricted to VEGFR-mediated angiogenic events. Nonetheless, VEGF-independent activation of VEGFR could be the result of indirect crosstalk between the G protein–coupled FPRs and VEGFR signaling [29], as previously suggested for retinal endothelial cells [30]. In RI-induced eyes, PAI-1/uPA/uPAR system is upregulated. In addition, TGFβ transcript levels were not associated with the mouse models of RI, in agreement with ECM degradation behavior mediated predominantly by a plasminogen-activator system. These observations again suggest higher involvement of uPAR/FPR signaling mechanisms over VEGFR-mediated pathways in iris neovascularization, demonstrating UPARANT as an ideal candidate for mitigation of RI.

Clinical treatment of NVG resulting from PR diseases, such as PDR and CRVO, currently centers upon anti-VEGF intravitreal injection [7]. Although clinical anti-VEGF agents reduce RI, the effects are limited; neovascularization may reoccur [8], and the needs for surgery and pan-retinal photocoagulation persist [31]. Such could be related to the fact that anti-VEGF treatments address exclusively VEGF signals, whereas a myriad of angiogenic factors and cytokines could be present in PR patients [3, 4]. The present data shows that intravitreal administration of UPARANT results in a sound reduction in neovascularization, even in VEGF-independent angiogenesis. UPARANT targets pathways upstream in the pro-angiogenic and pro-inflammatory cascades, thus downregulating a multitude of factors that mediate iris neovascularization and normalizing the iris vascular bed. The absence of VEGF signaling in the mouse model of RI contrasts with patients with chronic conditions, who present elevated VEGF levels in the eye. However, the ability of UPARANT to reduce VEGFR crosstalk signaling with G-coupled receptors [30], together with UPARANT’s effects on multiple transcription activators [12–15], contributes to the effectiveness of UPARANT as indicated in the RI mouse model. The broader mechanism of action could reveal UPARANT as an alternative for patients with ocular vasculopathologies that are resistant to anti-VEGF treatments. Furthermore, UPARANT demonstrated effectiveness upon systemic administration, which could impact clinical treatment of patients with proliferative ocular diseases such PDR and NVG.

## Electronic supplementary material

ESM 1 (PNG 4.23 mb)

High resolution image (EPS 121 mb)
