# Supplementary figures and images for: UPARANT is an effective antiangiogenic agent in a mouse model of rubeosis iridis
Source: J Mol Med (Berl). 2019 Jun 26;97(9):1273–83. doi: 10.1007/s00109-019-01794-w (PMC6713680; doi:10.1007/s00109-019-01794-w)

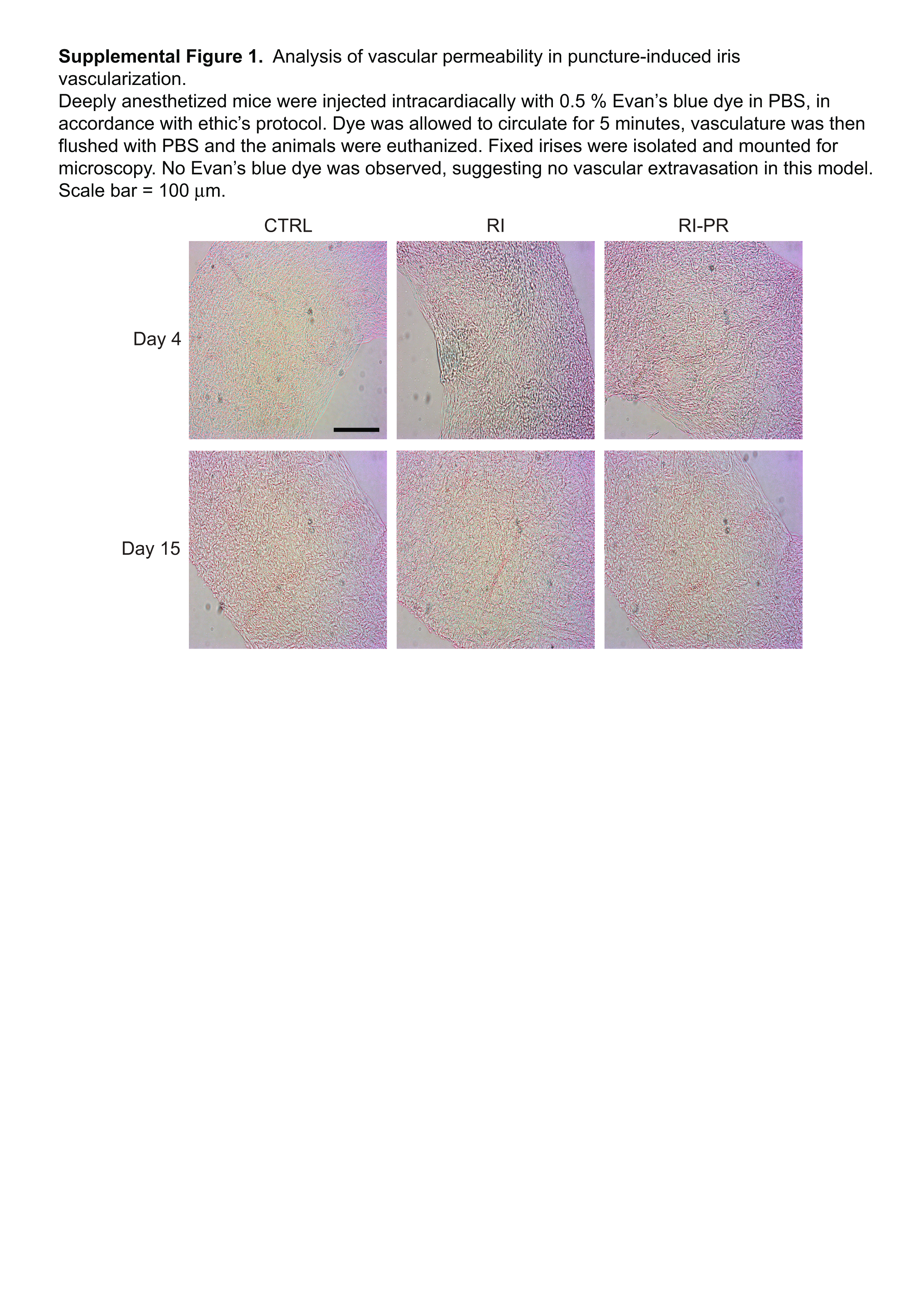

Supplement: Supplementary file 1 — (PNG 4.23 mb) [file 109_2019_1794_Fig7_ESM.png]
